# A Short Review on the Microstructure, Transformation Behavior and Functional Properties of NiTi Shape Memory Alloys Fabricated by Selective Laser Melting

**DOI:** 10.3390/ma11091683

**Published:** 2018-09-11

**Authors:** Xiebin Wang, Sergey Kustov, Jan Van Humbeeck

**Affiliations:** 1 Key Laboratory for Liquid-Solid Structural Evolution and Processing of Materials (Ministry of Education), Shandong University, Jingshi Road 17923, Jinan 250061, China; 2 School of Materials Science and Engineering, Shandong University, Jingshi Road 17923, Jinan 250061, China; 3 Departament de Física, Universitat de les Illes Balears, Cra Valldemossa km 7.5, E07122 Palma de Mallorca, Spain; Sergey.Kustov@uib.es; 4 ITMO University, Kronverkskiy av. 49, 197101 St. Petersburg, Russia; 5 Department of Materials Engineering, KU Leuven, Kasteelpark Arenberg 44, B3001 Heverlee, Belgium; Jan.Vanhumbeeck@kuleuven.be

**Keywords:** shape memory alloys, NiTi, additive manufacturing, selective laser melting, SLM, transformation behavior, functional properties

## Abstract

Due to unique functional and mechanical properties, NiTi shape memory alloys are one of the most promising metallic functional materials. However, the poor workability limits the extensive utilization of NiTi alloys as components of complex shapes. The emerging additive manufacturing techniques provide high degrees of freedom to fabricate complex structures. A freeform fabrication of complex structures by additive manufacturing combined with the unique functional properties (e.g., shape memory effect and superelasticity) provide great potential for material and structure design, and thus should lead to numerous applications. In this review, the unique microstructure that is generated by selective laser melting (SLM) is discussed first. Afterwards, the previously reported transformation behavior and mechanical properties of NiTi alloys produced under various SLM conditions are summarized.

## 1. Introduction

Near equiatomic NiTi shape memory alloys (SMAs) could appear in a B2 structured austenite (A), a B19’ structured martensite (M) or a rhombohedral R-phase, depending on the thermal or mechanical conditions [1]. The thermoelastic martensitic transformation between the abovementioned phases gives rise to the shape memory effect and superelasticity, which makes NiTi SMAs able to recover large deformations of up to 10% [2,3,4]. Due to the unique functional properties, together with the good biocompatibility [5], low stiffness [6,7], excellent corrosion resistance [8], high damping properties [9,10], and the excellent strength and ductility (tensile elongation >30% [11,12]), NiTi SMAs are the most promising functional metallic materials for practical applications in both medical (e.g., stents, guide wires) [13,14,15] and non-medical fields [16,17,18]. 

The demand for complex or customized NiTi SMA devices will increase remarkably in the future with the development of technologies in such fields as aviation and aerospace, personalized medical care, etc. However, fabrication of complex NiTi structures via conventional processing techniques (e.g., machining and welding) is difficult [19,20,21,22,23]. This difficulty is mainly caused by the high work hardening, high toughness, high strength, and high ductility (total tensile strain up to 70% [12]) of NiTi SMAs [24]. 

Unlike the conventional processing techniques, additive manufacturing (AM), which fabricates certain components by adding layers of materials progressively, provides great potential to produce complex or customized parts [25]. Various metal AM techniques, e.g., Selective Laser Melting (SLM), Laser Powder Deposition, and Wire Arc Additive Manufacturing, have been developed in the past decades [26,27,28]. Among the AM techniques, SLM generally provides a better surface finish and geometrical accuracy [25,28], which are the main features required for NiTi devices (e.g., stents, actuators). As a result, the fabricating of NiTi parts by SLM has frequently been addressed.

The work on additively fabricating the NiTi parts by SLM starts from producing dense and porous-free parts by optimizing the SLM process. The input laser energy density, which is determined by the SLM process parameters, is normally used as a guide to produce dense parts. The energy density (volumetric energy density, *E_V_*, and linear energy density, *E_L_*) can be simply estimated by:

*E_V_* = *P*/(*v* × *h* × *t*) (1)

*E_L_* = *P*/*v* (2)

where, *P*, *v*, *h*, and *t* represent the laser power, scanning velocity, hatch spacing, and layer thickness, respectively. The relative density of SLM fabricated NiTi parts benefits from the increase of energy density [29], like reported in other metallic materials [27,30,31,32,33,34,35], and a minimum energy density is required to produce fully dense (relative density > 99%) parts. As discussed in the work conducted by Haberland et al. [29], fully dense parts could be obtained when the energy density is higher than 200 J/mm^3^. The density of SLM produced parts will decrease slightly with an extra high energy density, due to the entrapping of gases, spatter, or improper closure of keyholes [33,36,37,38,39,40,41]. The optimization of SLM process to fabricate fully dense NiTi parts can refer to Ref. [24,29]. It is worth mentioning that various optimized energy densities for NiTi alloys have been reported in literature, from 55 to 300 J/mm^3^ [24,29,42,43,44,45,46,47,48,49]. An even higher energy density of 595 J/mm^3^ was used by Ma et al. [42], by reducing the hatch spacing. This indicates that many other factors have to be considered in order to optimize the SLM process, for instance, particle size, laser type, and spot size, as well as different combinations of SLM process parameters [50]. 

As compared with the samples that are produced via conventional approaches, the SLM fabricated parts show unique microstructure. On the other hand, the phase transformation behavior and functional properties, which are key factors affecting the practical applications of NiTi SMAs, are very sensitive to the change of microstructure [1,2]. Therefore, it is essential to understand the interrelation between SLM process and the resulted microstructure, and thus the phase transformation behavior and functional properties of the produced NiTi parts. In this review, the unique microstructure that is caused by SLM is discussed first. Afterwards, the previous works on explaining the phase transformation behavior, as well as improving the tensile properties of SLM fabricated NiTi parts, are summarized. 

## 2. Microstructure 

**Figure 1:**
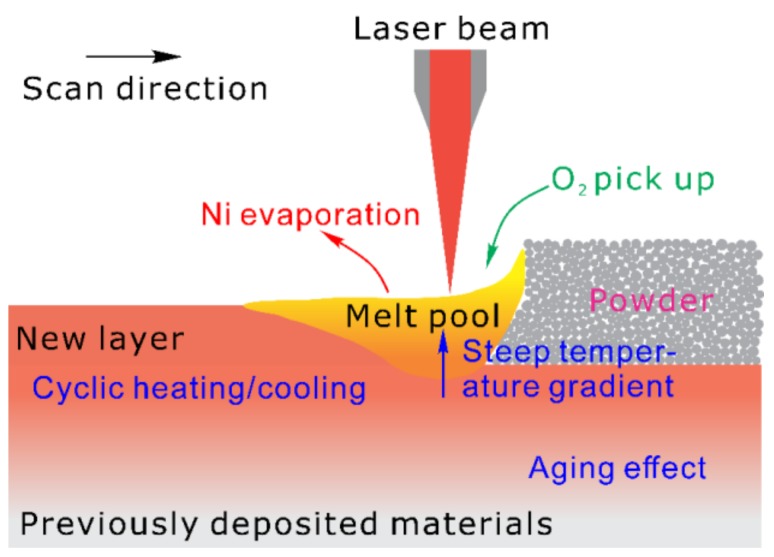
Schematic of the melt pool behavior of the selective laser melting process.

**Figure 2:**
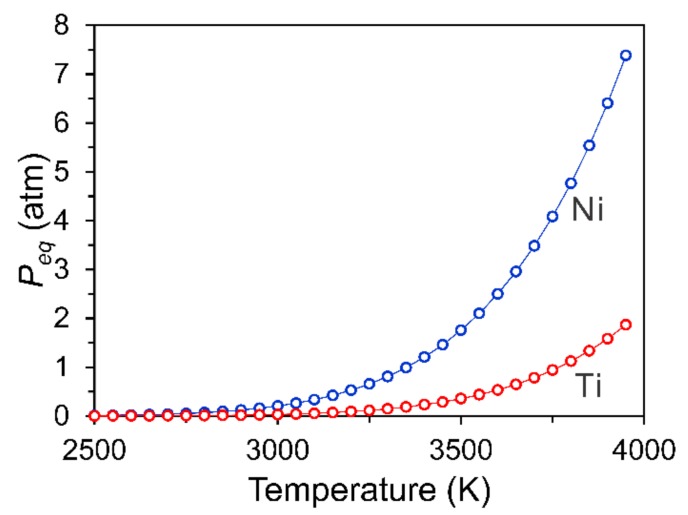
Equilibrium vapor pressures (*P_eq_*) of Ni and Ti over the liquid alloy for Ti-50.0 at % Ni alloy, which were calculated according to Ref. [54].

**Figure 3:**
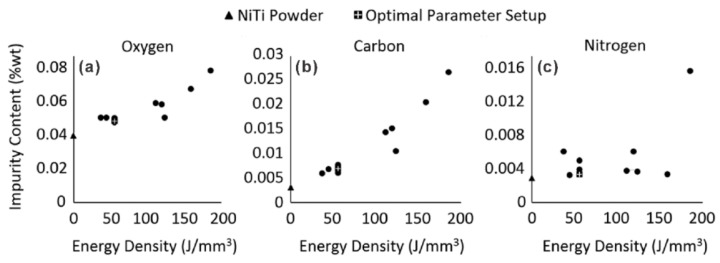
Oxygen, carbon, and nitrogen pickup in SLM fabricated NiTi parts [47] (with permission from SAGE Publications).

NiTi alloy powders are exposed to the laser beams with high energy density during SLM. The powders are heated up rapidly to a temperature above melting or even boiling points. When the laser beam moves away, the melt solidifies quickly due to the very high cooling rate (up to 10^6^ K·s^−1^ [51,52], depending on SLM process parameters and materials). This complex process, as shown schematically in Figure 1, repeats during SLM, and the previously solidified materials undergo a cyclic heating/cooling process. The unique thermal history leads to complex microstructural evolution during SLM, which affects remarkably both the transformation behavior and functional properties of the NiTi parts. The possible microstructural variation that is caused by SLM could be summarized, as follows:

(1) **Ni loss by evaporation**. Due to the high energy input from the laser, the evaporation of alloying elements from the melt pool will occur during SLM [42,53,54]. As shown in Figure 2, at elevated temperatures, the equilibrium vapor pressure of Ni is much higher than that of Ti, since Ni has a lower boiling point (3186 K) as compared with Ti (3560 K). As a result, the loss of Ni, i.e., decreasing of Ni/Ti ratio, will occur during SLM [55,56]. Moreover, an increase of Ni loss is expected with the increase of energy density [57]. It is well known that the transformation temperature of NiTi alloy depends highly on the Ni content [1,58]. Therefore, the Ni evaporation during SLM may lead to a remarkable increase of martensite transformation temperatures (MTTs). The melt pool behavior significantly affects the element evaporation, as discussed in the model that was developed by Klassen et al. [59]. Therefore, dedicated experiments or simulation work are highly required to study the Ni evaporation under different SLM conditions.
(2) **Oxygen pickup**. Since Ti is very active, pickup of oxygen will occur during SLM of NiTi alloys [45,47,60]. The oxygen pickup will lead to the following two effects: (1) binding with Ti (e.g., forming Ti_4_Ni_2_O [60,61,62]), resulting in an increase of effective Ni/Ti ratio and thus leading to the decrease of MTTs; (2) influencing significantly the mechanical properties of SLM fabricated NiTi parts. The latter effect depends on the size, morphology, and distribution of the oxides. Walker et al. [47] reported an increase of oxygen impurities with increasing energy density, as shown in Figure 3a. Therefore, it is essential to control the oxygen level in the building chamber to improve the repeatability in both the transformation behaviour and functional properties of SLM fabricated NiTi alloys. Many studies have shown that with proper process control (e.g., using the constant fresh argon flow during SLM [63]), the SLM fabricated NiTi parts show low oxygen content and meet the requirements (<500 ppm) prescribed in the ASTM F 2063 standard (ASTM International, Standard Specification for Wrought Nickel-Titanium Shape Memory Alloys for Medical Devices and Surgical Implants) [24,60,63,64]. The pickup of carbon and nitrogen also occurs during SLM, as shown in Figure 3b,c [47].
(3) **Precipitation**. During SLM, the fabricating parts are heated up due to the heat transferred from the melt pool and heat affected zone. In Ni-rich NiTi alloys, the precipitation of Ni_4_Ti_3_ phase may occur at a temperature as low as 473 K [65,66]. As a result, during fabricating Ni-rich NiTi parts, the formation of Ni_4_Ti_3_ precipitates may occur, which will significantly affect both the phase transformation behavior and mechanical performance of NiTi alloys. The Ni_4_Ti_3_ precipitation in SLM fabricated Ni-rich NiTi parts have been proposed in many studies [42,43,56,63,67]. Ni_4_Ti_3_ precipitates with the size <2 nm have been observed by means of high-resolution transmission electron microscopy [42,56]. As the presence of N_4_Ti_3_ particles significantly influences the performance of NiTi alloys [1], it is essential to study the size and distribution of Ni_4_Ti_3_ particles under different SLM process conditions. The formation of other precipitates e.g., Ti_2_Ni, was also reported [42,57,68,69].
(4) **Strong texture**. During SLM, the grains grow along the direction of the maximum temperature gradient, which is normally the same direction as the build direction (BD) [28]. The easy growth direction of the body centered cubic (BCC) crystals is <100> [28]. At elevated temperatures, near equiatomic NiTi alloys are in a B2 ordered austenite phase with BCC crystal structure [1]. Therefore, a strong <100>_B2_//BD (build direction) can be developed in SLM fabricated NiTi parts [43,67,70]. The strong texture will significantly influence the functional properties of NiTi alloys, as the transformation strain depends strongly on the crystallographic orientation [1]. It has been frequently reported that the texture characteristics (e.g., intensity or type of texture) depends highly on the SLM process conditions [28,71]. Therefore, it is suggested that future work is required to study the effect of the SLM process conditions on the texture characteristic of NiTi alloys and its influence on the functional properties.
(5) **High density of dislocations**. High density of dislocations could be introduced by SLM, due to the rapid cooling [42,72,73,74]. It has recently been reported that the dislocation network introduced by SLM could improve both the strength and ductility of the 316 L stainless steels, i.e., breaking the strength-ductility trade-off [72,73]. High density of dislocations has been reported in the SLM fabricated NiTi parts [42,56]. Moreover, the density of dislocations depends highly on the SLM process. For instance, Ma et al. [42] reported that the density of dislocations decreases with the decrease of hatch spacing from 35 to 120 μm, which probably is due to the recovery of dislocations that is caused by more re-melting and re-heating cycles when producing with smaller hatch spacing.
(6) **Residual stresses**. The locally melted metal is deposited on a relatively cold substrate (or previously consolidated layers), leading to a steep thermal gradient, which can surpass 10^7^ K·s^−1^ and 10^7^ K·m^−1^ [75]. As a result, the residual stress could be built up inside the SLM fabricated parts [28,76,77,78]. The accumulated residual stress can cause distortion, geometric failure, delamination of layers, deterioration of fatigue and fracture resistance, as well as the increase of anisotropy of the mechanical properties of SLM fabricated parts [28,76,77]. According to the Clausius-Clapeyron type dependence of MTTs on stress [79], the accumulation of residual stresses will assist the martensite transformation.
(7) **Inhomogeneous grain size distribution**. Due to the complex thermal history, the microstructure with inhomogeneous grain size distribution is normally observed in the SLM fabricated parts [57,71,80,81]. The mechanical performance will be affected remarkably by the inhomogeneous microstructure [81,82,83].
(8) **Microstructural heterogeneity**. The materials at different position of SLM fabricated parts will experience a different thermal history. For instance, the first deposited layers will experience a fast cooling due to the cold substrate, as well as more reheating cycles, as compared with the top layers. Therefore, the heterogeneous microstructure (e.g., inhomogeneous Ni distribution, thermal stress state, grain size) is developed in the SLM fabricated parts [24,84]. The microstructural heterogeneities will significantly affect the transformation behavior and mechanical properties of SLM that are produced NiTi parts [84].

## 3. Phase Transformation Behavior

The “Martensitic Transformation Temperatures (MTTs)” are critical factors affecting practical applications of NiTi alloys, as they determine the temperature range on which shape recovery occurs. The transformation behavior of NiTi alloys is very sensitive to microstructural changes (e.g., presence of dislocations or precipitates). SLM process gives rise to a unique microstructure, being strongly affected by SLM process parameters. Therefore, it is essential to understand how the transformation behavior of NiTi parts reacts to the variations of the SLM process parameters.

As compared with starting NiTi powders, the SLM fabricated parts normally show higher transformation temperatures [29,49,62,85,86], an example could be found in Figure 6 of Ref. [29]. The increase of transformation temperatures is likely due to the Ni evaporation during the SLM process [29,47,56,87,88]. It seems that with a high Ni-rich starting powder (e.g., Ti-50.7 at % Ni), the shift of transformation temperatures is more obvious than that with a less Ni-rich powder (e.g., Ti-50.2 at % Ni) [29,63]. Therefore, it is important to carefully select the composition of the starting powders, in order to produce NiTi parts with the desired functional properties at the envisaged temperature range. 

It has frequently been reported that MTTs increase with the increase of energy density [24,29,42,48,56,57,63,87,89]. Moreover, this increase of MTTs is more obvious in the Ni-rich samples than in the Ti-rich samples [63]. The precipitation of Ni-rich Ni_4_Ti_3_ particles is also a possible reason for the increase of MTTs [42], because the formation of Ni_4_Ti_3_ particles leads to Ni-depletion from the matrix [1,2]. However, the Ni_4_Ti_3_ precipitates are normally very small in SLM fabricated NiTi alloys [42,56], and nano-sized Ni_4_Ti_3_ precipitates indeed suppress martensite transformation (MT), instead of promoting MT, due to the intense lattice distortion that is caused by the coherency between Ni_4_Ti_3_ and B2 matrix [90,91,92]. 

Therefore, the Ni loss that is caused by evaporation is likely the main reason for the increase of MTTs. It is suggested that more Ni is evaporated with the increase of energy density, leading to the decrease of Ni/Ti ratio and thus the increase of MTTs. Similar results have been reported in laser welding of NiTi alloys [20,93,94]. Zamani et al. [93] found that the MTTs increase with the increase of laser power, indicating the increase of Ni loss with the increase of input energy. Oliveira et al. [94] reported that the MTTs increase gradually from base materials to the weld centreline, which can be attributed to the increase of Ni loss with the increase of laser energy, as the laser energy increases from heat affected zone to the center of the melt pool.

However, it was frequently observed that the MTTs differ largely between samples fabricated under similar energy density, but with different SLM process parameters [43,45,46,49], indicating that the physics behind the observed phenomenon in SLM fabricated NiTi alloy is rather complex. Moreover, it was also reported that the samples, which were produced with very low energy density, show a lower transformation temperature than the MTTs of the powders [29,63]. Therefore, the Ni loss by evaporation is not the only reason for the variation of MTTs with respect to the change of energy density (or SLM process parameters).

**Figure 4:**
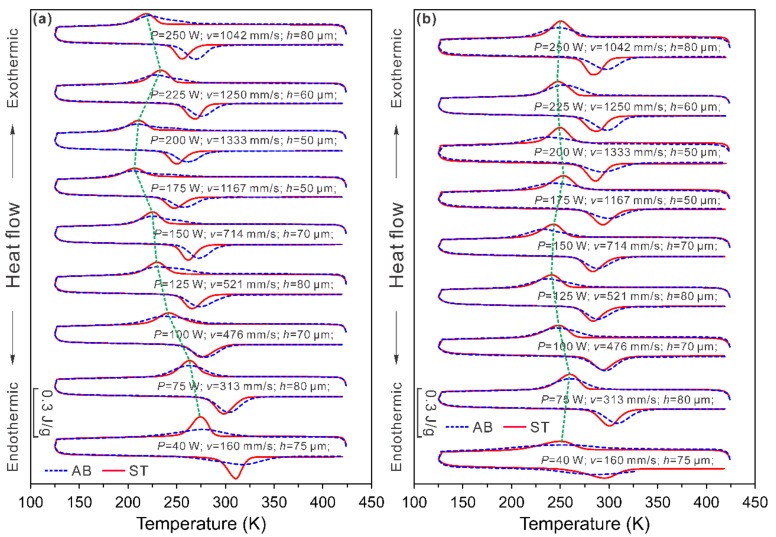
DSC (Differential Scanning Calorimetry) curves of the as-built (AB) and solution treated (ST, 1273 K for 2 h) NiTi samples produced by various SLM processes under (**a**) high O_2_ and (**b**) low O_2_ conditions. The green dash line guides the transformation peaks of the solution treated samples. *P*, *v*, and *h* represent the laser power, scanning velocity, and hatch spacing, respectively. (Modified after [45]).

In our previous work [45,46], the variation of MTTs was also observed in the samples produced with the same energy density (100 J·mm^−3^) but different process parameters, as shown in Figure 4a. The MTTs, which are defined as the temperature of the transformation peaks (*Mp*), change from 205 to 277 K under different SLM process conditions. Solution treatment (1273 K for 2 h) reduces the transformation interval for all the samples, but the temperatures of the transformation peaks remain essentially unaffected. This indicates that the variation of MTTs is mainly due to the modification of Ni/Ti ratio, instead of the presence of precipitates, internal stress, or dislocations, because the solution treatment could dissolve the Ni_4_Ti_3_ precipitates, annihilate dislocations, as well as eliminate largely the internal stresses. 

Figure 4b shows another set of samples, which were produced with the same SLM process parameters as the samples in Figure 4a, but under a lower oxygen level. As compared with the samples produced under high oxygen level (Figure 4a), a much narrower variation of MTTs (between 233 and 261 K) was observed [45]. This indicates that the oxygen pickup remarkably influences the transformation behavior of the SLM fabricated NiTi parts. Therefore, it is essential to control the oxygen level of the chamber during the SLM process.

By comparing the transformation behaviour of the samples before and after solution treatment (1273 K, 2 h), we could conclude that the variation of MTTs of NiTi samples that were produced under different SLM conditions is mainly due to the modification of effective Ni/Ti ratio. Feasible reasons for the modification of Ni/Ti ratio are: (i) Ni evaporation, which decreases the Ni/Ti ratio; (ii) oxygen pickup, which binds Ti and thus results in an increase of the effective Ni/Ti ratio; and, (iii) formation of Ni_4_Ti_3_ precipitates changing the Ni/Ti ratio of the matrix. The competition between the above effects determines the variation of MTTs. 

It is rather difficult to quantify the Ni loss or oxygen pickup in experiments, because these characteristics are influenced by many factors, e.g., melt pool size, the maximum temperature of melt pool, the exposure time of the melts to atmosphere, and the oxygen content of the chamber. Therefore, simulation work, e.g., the model proposed by Khairallah et al. [37], is highly relevant to reveal the relation between SLM process and the loss of Ni or Ti. 

The precipitation of Ni_4_Ti_3_ particles, presence of dislocations and residual stresses, as well as the microstructural heterogeneity also affect the transformation behavior (e.g., multiple transformation peaks, broadening transformation peaks) of as-fabricated NiTi parts. Thus, the effect of solution treatment and subsequent aging treatment on the transformation behavior and the mechanical properties of SLM fabricated NiTi parts are also worth studying.

## 4. Tensile Properties

One of the most challenging tasks in SLM of NiTi SMAs is to produce NiTi parts with good functional and mechanical properties. This is currently the main issue that hampers the practical application of SLM fabricated NiTi parts. 

Many studies, which were mainly focused on the compression mode, have been conducted to investigate the mechanical properties of SLM fabricated NiTi parts [24,29,43,47,49,60,62,67,68,70,85,95,96,97,98,99]. The effect of SLM process conditions on the functional properties of NiTi alloys under compression mode has been well studied and summarized, for example in Ref. [24,43,100]. The SLM fabricated NiTi parts normally show good mechanical properties under compression. The total compressive strain over 25% [60,63,98] and recoverable strain of up to 6% [43] have been reported, which are comparable to the conventionally fabricated NiTi samples. It was also found that the compressive properties of SLM fabricated NiTi alloys also benefit from aging treatment [24,70,101], the effect that is similar to that in NiTi produced by conventional techniques [65,90].

Besides compression, the tensile properties of NiTi parts are also important, because many NiTi devices work under tension or tension-distortion conditions. However, only very few studies tested the performance of SLM fabricated NiTi parts under tension [56,57,100,102] or bending [42,103]. Tensile properties are more sensitive to the defects generated by SLM (e.g., porosity, microcracks), leading to the low fracture strains and stresses under tension.

**Figure 5:**
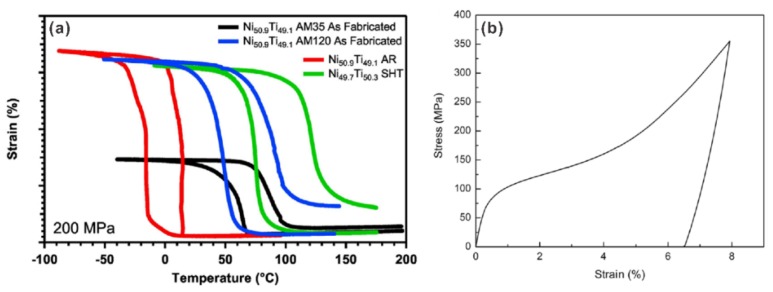
(**a**) Isobaric heating-cooling curves of a Ni_50.9_Ti_49.1_ alloy fabricated with a small hatch spacing of 35 μm (black curve), and a large hatch spacing of 120 μm (blue curve). For both samples, the laser power (50 W), scanning sapped (80 mm s^−1^), and layer thickness (30 μm) were the same. The results of the conventionally fabricated Ni_50.9_Ti_49.1_ (red curve) and a Ni_49.7_Ti_50.3_ alloy after solution treatment (900 °C, 1 h, green curve) are also shown [56] (with permission from Elsevier Publisher); (**b**) Stress-strain curve of a NiTi ribbon fabricated by repetitive laser scan. A laser power of 25 W was used for the first scan, and the laser power of 60 W was used for the second scan [100] (with permission from Springer Nature).

Recently, Sam et al. [56] investigated the tensile properties of NiTi samples fabricated with different energy densities by modifying the hatch spacing. A recoverable strain of around 5% has been obtained in the samples that were produced with certain SLM parameters (Figure 5a) [56]. It was also found that the sample fabricated with low energy density (with the hatch spacing of 120 μm) shows a larger recoverable strain than that fabricated with high energy density (with the hatch spacing of 35 μm) [56]. The detailed reason for the improvement of the tensile performance with a larger hatch spacing is not clear yet. It is suggested that future work is highly required to study the influence of SLM process conditions on the microstructure and thus the mechanical properties of NiTi alloys. Khoo et al. [100] reported a NiTi ribbon with transformation strain (under tension) of 4.6% (Figure 5b). The ribbon was fabricated by repetitive laser scanning, i.e., (partially) re-melting the previously deposited layer. It seems that re-melting could eliminate the defects (e.g., cracks, pores) and thus improve the tensile properties. However, only one layer was fabricated in this study. Future studies are required to investigate the effect of re-melting on the performance of SLM fabricated bulk parts.

In the work conducted by Hayat et al. [104], NiTi parts were fabricated while using Selective Electron Beam Melting (EBM), which offers a high vacuum circumstance during processing, and thus leads to low pick-up of O_2_ and other impurities. Moreover, a preheating temperature of 750 °C kept during processing. However, the EBM fabricated sample shows only the tensile strain of 3.9%. This indicates that the contamination of O_2_ may be not the main reason for deteriorating the tensile properties of NiTi alloys fabricated by SLM. Therefore, detailed research (e.g., in-situ TEM work) on finding the origin of the brittleness of SLM fabricated NiTi alloy is highly required, especially to compare the microstructure with the NiTi alloys that were fabricated via conventional approaches.

SLM gives rise to a unique microstructure, which will significantly affect the functional performance of NiTi parts. For instance, the strong texture leads to high anisotropy in the functional properties in different directions (with reference to the build direction). This has to be considered especially for the NiTi devices, which are designed for deforming at different directions. The pickup of oxygen and other impurities, and the presence of pores and micro-cracks will undermine the functional performance of NiTi parts, especially under tension. The inhomogeneous microstructure that is caused by SLM may lead to unpredictable mechanical behavior, as compared with the conventionally fabricated NiTi parts. It is suggested that future work is required to understand how the unique microstructure affects the functional performance of SLM fabricated NiTi parts. 

## 5. Conclusions

In this brief review, previous studies on fabricating NiTi parts by SLM were summarized. SLM provides great potential for fabricating geometrically complex NiTi devices, which will significantly promote the practical applications of NiTi SMAs. SLM gives rise to a unique microstructure, which is highly sensitive to the change of SLM process parameters. The transformation behavior and functional properties of NiTi alloys are, in turn, very sensitive to the microstructural changes. Therefore, more systematic studies are necessary to establish the relation between the SLM process and resulting microstructure, as well as the functional performance of NiTi parts.

(1) SLM is a complex physical metallurgical process, which leads to the complex microstructural changes, including the variation of composition, formation of precipitations and dislocations, development of strong texture and residual stresses, and the microstructural heterogeneity. Systematic work is required to study the interrelationship between SLM process and resulted microstructure and related functional properties.
(2) The phase transformation behavior are very sensitive to the SLM process conditions, even when fabricating under similar energy density level. Although the interrelationship between the SLM process and the transformation behavior of NiTi alloys is not clear yet, it may provide an effective way to tailoring the transformation temperature of NiTi alloys by tuning the SLM process parameters.
(3) The compression properties of SLM fabricated NiTi alloys are comparable with the NiTi alloys produced via conventional approaches. However, the SLM fabricated NiTi alloys normally show a total elongation < 6% under tension. The origin of the brittleness of SLM fabricated NiTi alloy is not clear yet. Future work is highly required to study the formation of defects (e.g., voids, micro-cracks) under different SLM process conditions, and their influence on the functional performance of SLM fabricated NiTi alloys.
